# Surgical Management of Post Burn Hand Deformities

**DOI:** 10.12669/pjms.36.6.2206

**Published:** 2020

**Authors:** Suneel Kumar, Faisal Akhlaq Ali Khan, Hyder Ali, Saba Kiran

**Affiliations:** 1 Suneel Kumar, FCPS-2, Post Graduate Trainee of Plastic Surgery. Department of Plastics and Reconstructive Surgery, Dow University of Health Sciences, DR. Ruth KM Pfau, Civil Hospital, Karachi, Pakistan; 2 Faisal Akhlaq Ali Khan, Chairperson and Assistant Professor, Head of Department of Plastic Surgery. Department of Plastics and Reconstructive Surgery, Dow University of Health Sciences, DR. Ruth KM Pfau, Civil Hospital, Karachi, Pakistan; 3 Hyder Ali, Assistant Professor of Plastic Surgery. Department of Plastics and Reconstructive Surgery, Dow University of Health Sciences, DR. Ruth KM Pfau, Civil Hospital, Karachi, Pakistan; 4 Saba Kiran, FCPS-2, Post Graduate Trainee of Plastic Surgery. Department of Plastics and Reconstructive Surgery, Dow University of Health Sciences, DR. Ruth KM Pfau, Civil Hospital, Karachi, Pakistan

**Keywords:** Burn injury, hand injury, loco-regional flaps post burn hand contractures, surgical management, skin grafts

## Abstract

**Objective:**

To evaluate the efficacy of different surgical procedures on post burn contracture of hand.

**Methods:**

A quasi-experimental study design was conducted at the Department of Plastics and Reconstructive Surgery, Dow University of Health Science, DR. Ruth KM Pfau, Civil Hospital, Karachi, Pakistan from 1^st^ June 2019 to 30^th^ November 2019. Ninety-three participants of burned hand contracture of either gender, aged between 6- 60 years were included in the study. Resurfacing surgery with skin graft and loco-regional flaps were done according to type of contracture with individualization for each patient. All patients were kept under follow up for ninety days to assess efficacy of contracture release for each surgical procedure was noted. SPSS version 23 was used to analyse data.

**Results:**

Full thickness skin graft (FTSG) was performed in 60.2% cases, 17.2% with split thickness skin graft (STSG) and 12.9% with cross finger flaps. About 25% of recurrence was observed in cross finger flaps, whereas no recurrence was seen in Z-plasties and posterior interosseous flap. The significant association was between recurrence and surgical procedures (p<0.05).

**Conclusion:**

Z-plasty followed by FTSG was effective in the management of post burn contractures of hand.

## INTRODUCTION

Globally, burn injury is the 2^nd^ leading cause of trauma-related mortalities.^1^ In South-East Asia, about 40% of all death is induced by burns.^1^ Deep burns if improperly managed can lead to post-burn hand contractures.^2^ Post-burn contractures are one of the main complications of burning accidents aside from tissue and skin defects.^3^ The hands are the most frequently burnt site. Literature showed that approximately 39% of burn injuries occurred in upper extremity and hands.^4^

Burned patients usually experienced deformity, scarring and several psychosocial symptoms such as pain or pruritus.^5^ Post burning of the hands in particular may have an effect on the everyday work and aesthetic appearance of patients. Management of such patients therefore should be first to improve function particularly in essential regions of the body, including the hand.^6^ Hand contractures can be avoided by means of prompt proper care like hand elevation, proper splits application and grafting of burn area followed by rehabilitation of hand.^7^ There is great variety of surgical option are available such as skin grafting after releasing scars along with k- wire fixation to provide immobilization for graft take, skin grafting gives advantage of budget, time and its availability.^5^ Previous study showed that split thickness skin grafts (STSG) and full thickness graft (FTSG) reconstructed cases had good recovery of joint mobility in 43% and 75% of cases respectively. Reconstructive procedures were aesthetically acceptable to the patients in 63%, 75% and 94% of STSG, FTSG and Z plasty cases respectively and recurrence was seen in 17% of STSG done cases.^8^

We are facing a massive number of patients suffering from contractures of burnt hands in our population. The objective of this study was to evaluate the efficacy of different surgical procedures used to correct deformity. This study would add the data in medical institute which would be supportive in choosing the better treatment plan.

## METHODS

It was a quasi-experimental study conducted at the Department of Plastics and Reconstructive Surgery, Dow University of Health Science, DR. Ruth KM Pfau, Civil Hospital, Karachi, Pakistan from 1^st^ June 2019 to 30^th^ November 2019. A total of 93 patients of age 6 to 60 years of either gender with burned hand contracture (McCauley’s Grades III and IV)^9^ were included using non-probability purposive sampling method. Patients with traumatic or bilateral or recurrent hand contractures or patients who were unfit for surgery were excluded from the study.

Ethical approval of the study was obtained from Ethical Review Committee (Ref: IRB-1291/DUHS/Approval/2019 Dated: June 1, 2019) of Dow University of Health Sciences, DR. Ruth KM Pfau, Civil Hospital, Karachi. Informed written consent was taken from all the eligible patients. The purpose, benefits and potential risks were explained to the patients. The demographic information was collected from all the patients.

The detail medical history and physical examination was carried out and pre-operative assessment of hand deformity was performed and scored on the basis of Disabilities of the Arm, Shoulder and Hand (DASH) Score (%). Grading of burn was done according to McCauley^9^ and based on the location of scar, involvement of skin, tendon, joints and bone an appropriate surgical option was chosen. All patients were operated under general anaesthesia after applying all aseptic measures and draping protocol. Finger contractures were released and at the corner fish tail extension release was given, the neurovascular bundles and underlying tendons were not exposed during release were resurfaced with skin grafting. Those cases had exposed vital structures were covered with cross finger flaps and other loco-regional flaps. Skin grafts were used to cover secondary defects. For the web space correction, we had been used the z-plasties, specifically the 1^st^ web spaces. K-wire fixation along with back slab was provided in all patients for three weeks, thereafter physiotherapy, oil massage and nighty splint was described for 3 months. All patients were kept under follow up for ninety days and during each follow-up visit DASH score was noted. The change in DASH score after each procedure was assessed by comparing the pre-operative DASH score and post-operative DASH score at 90^th^ day. The efficacy was labelled as positive when there was no recurrence at 90^th^ day.

All the collected data was analysed through SPSS version 23. Mean and SD was reported for numeric variables, whereas frequency and percentage was reported for qualitative variables. Paired t-test was used to compare pre and post DASH score of each surgical procedure. Chi-square/Fisher exact test was applied to assess the association between efficacy (no recurrence) and demographic variables and procedures. P-value≤0.05 was taken as statistically significant.

## RESULTS

This study was conducted on 93 patients of hand deformities secondary to burns. All patients were treated surgically and then followed up for next ninety days. The mean age of patients was 27.36 years and majority of patient were in the age range of 31-45 years (34.4%). Male patients were 62 (66.7%) whereas the female patients were 31 (33.3%). Seventy nine (84.9%) patients were right hand dominant whereas 14(15.1%) patients were left hand dominant. These patients belonged to different occupations and was grouped as students (n=24, 25.8%), laborer (n=47, 50.5%), house wife (n=13, 14%) and others (n=9, 9.7%) respectively.

Full thickness skin grafts (FTSG) was performed in 56 cases (60.2%) followed by split thickness skin graft (STSG) (17.2%), cross finger flaps (12.9%), Z-plasties (8.6%) and posterior interosseous artery flap (PIA) (1.1%). The significant decrease in DASH scores was observed in patients who had STSG, FTSG, cross finger flap and Z platies (p<0.05). Only one patient had PIA flap and DASH score increased post procedure. (Table-I)

**Table-I:** Comparison of mean DASH score pre-operative and post-operative (n=93).

Surgical procedures		Pre	Post	P-value

	n	Mean±SD	Mean±SD	
STSG	16 (17.2%)	26.20±5.45	9.85±2.21	0.001
FTSG	56 (60.2%)	25.21±6.99	8.68±2.29	0.001
Cross Finger Flap	12 (12.9%)	24.16±6.37	8.85±1.72	0.003
Z plasties	8 (8.6%)	24.34±3.91	9.53±2.23	0.008
PIA Flap	1 (1.1%)	31.02	38.26	-
Total	93 (100%)	25.24±6.37	9.30±3.76	0.001

The overall skin grafting results are satisfactory (70%) but loco-regional flaps like z-plasty and cross finger flaps showed better efficacy. In age group 13-18 years, no recurrence of contracture was seen in 91.7% cases. Among males 82.3% showed no recurrence whereas 81% efficacy was observed in right handed patients. Statistically there was no association between recurrence and age, gender and dominant hand (p>0.05). Despite of k-wire fixation and external application of back slab the recurrence was seen in 37.5% cases those who had STSG, 25% cases who had cross finger flaps and 16.1% cases who had FTSG, whereas no recurrence was seen in Z-plasties flap. None of our patient had infection of resurfaced or either donor area was noted. There was partial loss of split thickness graft in two cases and loss of one posterior interosseous artery flap was encountered. The significant association was between recurrence and surgical procedures (p<0.05). (Table-II)

**Table-II:** Distribution of recurrence of post burn contractures in different surgical procedures.

Characteristics	Recurrence		Non recurrence		P-value

	n	%	n	%	
***Age***					0.180
6 – 12	5	33.3	10	66.7	
13 – 18	1	8.3	11	91.7	
19 – 30	3	11.1	24	88.9	
31 – 45	7	21.9	25	78.1	
46 – 60	3	42.9	4	57.1	
***Gender***					0.363
Male	11	17.7	51	82.3	
Female	8	25.8	23	74.2	
***Hand dominance***					0.412
Right handed	15	19.0	64	81.0	
Left handed	4	28.6	10	71.4	
***Surgical procedures***					0.047
STSG	6	37.5	10	62.5	
FTSG	9	16.1	47	83.9	
Cross finger flaps	3	25.0	9	75.0	
Z Plasty	0	0.0	8	100.0	
PIA Flaps	1	100.0	0	0.0	

## DISCUSSION

Hand includes less than 5% of surface area of body.^10^ Plastic surgeons routinely face the patients of finger burn deformities.^11^ The overall management of such type of deformities takes longer time, complex surgical techniques^12^ and remain a challenging mission in the plastic surgery arena.^13^ The contracture release is not only indicated for functional drive but also requisite for aesthetics.^14^ Therefore, current study was conducted to evaluate the efficacy of different surgical procedures used to correct deformity. This study would add the data in medical institute which would be supportive for plastic surgeons in choosing the better treatment plan.

In this study, mean age of the patients was 27.36 years and majority of them were males and had right hand involved in bury injury. In the study by Sunil et al. found the mean of the patients as 14 years and 54% were males and three patients were being operated for contracture of both hands.^8^ However it has been seen in literature that females are at higher risk of burns due to their exposure to open fire cooking, or inherently unsafe cooking stoves, or domestic violence. Moreover, children are particularly at greater odds of burning due insufficient adult supervision and maltreatment.^15^ Dissimilarity in our findings may be due to occupations of males that increases their exposure to fire.

In this study we can see significant decrease in DASH scores in patients who had STSG, FTSG, cross finger flap and Z platies (p<0.05). In a previous study by Mohammed H et al. 27 adult 2nd degree burned patients showed gradual improvement of the upper limbs’ function measured through DASH scale after application of physiotherapy protocol, this appear through significant decrease in complain from severe dysfunction among 25.9% after 2 months to 0% after 6 months.^16^ These findings highlight that the upper limbs’ function post burns could be improved by preventive nursing interventions including early range of motion, anti-contracture positioning and splinting of hand.^16^

We also noted that children aged 6-12 years and adolescents aged 13-18 years showed satisfactory outcomes of surgical procedures as compared to adults aged more than 18 years. In children contractures released were better resurfaced with full thickness skin grafts due to reduced secondary contraction compared with split thickness skin grafts. There are certain limitations of full thickness graft like providing insufficient graft for bigger wounds, hairy grafts and lower graft take rates. Whereas literature showed that the split thickness grafts have early take rate, reduced infection rates and used to resurface larger areas but has some disadvantages such as increased count of secondary contractures and cosmetically less pleasant.^17^ Full thickness skin graft take is also influenced by infection due to its reduced rate of graft take.^18^

Evidence showed that split thickness skin graft, was an effective way of providing active range of motion and cosmetically acceptable hand.^19^ Results of Chan et al comparison study was in favor of full thickness skin grafts but there was no difference was found in the recurrence rate among two groups.^20^ Whereas in the current research, Z-plasty showed 100% efficacy and FTSG was 83.9% effective. Few authors had used prepuce as skin grafting, had some benefits over skin grafts donor areas like it is pliable, thin, decreased tendency to shrink, adequate colour match and take, whereas as few disadvantages were seen in this study was the hyperpigmentation noted in few cases. This study only confined to male patients, contracture involving fingers exceeds three digits were not suitable candidates and was not applicable on patients those who had underwent circumcision before burns.^21^

Pensler et al. conducted a comparison study on palm contractures in children in which he had compared the results of full thickness versus split thickness skin graft after releasing palm contractures but there was no functional change seen in his study.^22^ Some author have utilized the Joshi external stabilization system (JESS) after releasing burn contractures and resurfacing it with split thickness skin grafts. The reoperation rate was 40% which was comparable with other studies.^23^ As a general principle, patients who had operation had mature contractures because of the risk of later on recurrence. Each scar was thoroughly assessed for scar maturity like soft, supple and non-blanch able. Tissue utilized to correct deformity depends upon varying degree of severity and location of defect. The use of only skin grafting in the web spaces was not beneficial due recurrence of deformity, Z-plasty was beneficial in web space and linear bands release, specifically in first web space. The cross finger flap was utilized in those case in which during release the finger tendon and neurovascular structure were got exposed, this flap provides thick supple tissue from the same region and prevents later on recurrence. This technique had certain disadvantages like it need a donor site graft, need second surgery for division of flap.

The defect found after releasing burn contracture was resurfaced by five diverse modalities was questionable. Most of our patients had simple skin contractures involving fingers, full thickness and split thickness skin grafting was used in bulk of patients compared to loco regional flaps, despite of complete release of contracture and available physiotherapist in our plastic surgery department the non-compliance and poor follow up of rehabilitation protocol was noted in recurrent cases. Whereas other patient with compliance and proper follow up had achieved adequate hand drive, aesthetically acceptable hand and had better quality of life. This study will be adding data in medical institute which can be beneficial in option the better treatment planning. There are certain limitations about study are small sample size of patients, single centre study and no randomization was done. Further prospective studies and randomization will help decreasing the limitations.

## CONCLUSION

Post-burn contractures are the common outcomes of burn injury that should be avoided by selecting the adequate prevention and management strategies. In most of the cases Z-plasty followed by FTSG was the adequate treatment. However, further physiotherapy and splinting is needed to restore complete hand functions. Therefore, apart from surgery the patient compliance and the proper follow up of rehab protocol should be considered as pivotal parameter.

### Authors’ Contribution:

**SK:** Conceived idea, manuscript writing. He is also the responsible and accountable for the accuracy or integrity of the study.

**FAAK:** Statistical analysis, proof reading.

**HA:** Literature searching, contribution in manuscript writing.

**SK:** Data collection, critical review.
